# Caveolin-1 influences human influenza A virus (H1N1) multiplication in cell culture

**DOI:** 10.1186/1743-422X-7-108

**Published:** 2010-05-26

**Authors:** Lijing Sun, Gun-Viol Hemgård, Sony A Susanto, Manfred Wirth

**Affiliations:** 1 Division of Molecular Biotechnology, Helmholtz-Centre for Infection Research, Inhoffenstr. 7, 38124 Braunschweig, Germany; 2 National Key Laboratory of Biochemical Engineering, Institute of Process Engineering, Chinese Academy of Sciences, No.1 Bei-er-tiao, 100080 Beijing, China; 3 Graduate University of Chinese Academy of Sciences, 19A Yu Quan Rd, 100049 Beijing, China

## Abstract

**Background:**

The threat of recurring influenza pandemics caused by new viral strains and the occurrence of escape mutants necessitate the search for potent therapeutic targets. The dependence of viruses on cellular factors provides a weak-spot in the viral multiplication strategy and a means to interfere with viral multiplication.

**Results:**

Using a motif-based search strategy for antiviral targets we identified caveolin-1 (Cav-1) as a putative cellular interaction partner of human influenza A viruses, including the pandemic influenza A virus (H1N1) strains of swine origin circulating from spring 2009 on. The influence of Cav-1 on human influenza A/PR/8/34 (H1N1) virus replication was determined in inhibition and competition experiments. RNAi-mediated Cav-1 knock-down as well as transfection of a dominant-negative Cav-1 mutant results in a decrease in virus titre in infected Madin-Darby canine kidney cells (MDCK), a cell line commonly used in basic influenza research as well as in virus vaccine production. To understand the molecular basis of the phenomenon we focussed on the putative caveolin-1 binding domain (CBD) located in the lumenal, juxtamembranal portion of the M2 matrix protein which has been identified in the motif-based search. Pull-down assays and co-immunoprecipitation experiments showed that caveolin-1 binds to M2. The data suggest, that Cav-1 modulates influenza virus A replication presumably based on M2/Cav-1 interaction.

**Conclusion:**

As Cav-1 is involved in the human influenza A virus life cycle, the multifunctional protein and its interaction with M2 protein of human influenza A viruses represent a promising starting point for the search for antiviral agents.

## Background

In the last few years the interaction of viral matrix proteins or precursors with cellular proteins has attracted much attention in the field of medical virology due to the increase in the understanding of their interplay in late viral processes like protein transport, virus assembly and budding. Viral matrix proteins establish the link between outer shell and capsid core of enveloped viruses and bring together these parts in the virus assembly step. Moreover, matrix proteins frequently determine the place where the assembly step occurs. In influenza A viruses two M proteins are located on RNA7 of the negative-stranded, segmented RNA virus. The M1 protein functions as a typical matrix protein, while M2 exerts multiple tasks in the early and late phase of virus infection. M2 tetramers form an ion channel and in the early phase of virus infection M2 serves for the release of viral nucleocapsid by acidification of endosomes. In the late phases, M2 prevents premature activation of newly synthesized HA [1] and -in concert with M1- contributes to virus budding and morphology. The involvement in virus exit has been assigned to the cytoplasmic tail of the protein [2-4]. Influenza viruses bud from lipid rafts and for this event the components of the viral envelope (haemagglutin HA, neuraminidase NA, M2) and the RNA containing protein complex (vRNP) must come together to form infectious virus [5-7]. Interestingly, the endosomal sorting machinery (ESCRT), which has been involved in late steps of other viruses, does not contribute to influenza virus budding [6,8]. Accordingly, other routes and gates have been suggested for the transport of influenza proteins and virus assembly/budding [5].

In several previous investigations caveolin-1 (Cav-1), a multifunctional, raft-resident membrane protein has been linked to the virus replication of retroviruses HIV-1 and amphotropic mouse leukemia virus, rotavirus and respiratory syncytial virus [9-13]. Interestingly, a contribution of Cav-1 to HA transport has been reported for influenza virus infected MDCK cells [14]. In a recent investigation of the enveloped γ-retroviruses budding from lipid rafts we showed that caveolin-1 (Cav-1) interacts specifically with the MLV retroviral matrix protein in the Gag precursor, suggesting that Cav-1 serves in positioning the Gag precursor at lipid rafts [13]. Not surprisingly, Cav-1 is incorporated into MLV virions released from mouse NIH3T3 [13,15]. Subsequently, competition and inhibition experiments provided evidence that Cav-1 modulates MLV retrovirus production [13]. Taken together, these findings pointed to a general contribution of Cav-1 in virus replication strategy and opened the possibility that other virus families budding from lipid rafts may co-opt the functions of Cav-1. In our search for cellular/viral targets a database screen for Cav-1 binding sites notably revealed that structural proteins like matrix proteins of other viral families, e.g. *Orthomyxoviridae *with influenza A virus as a representative, exhibit regions of homology with a consensus motif for Cav-1 binding (Cav-1 binding domain, CBD) (Wirth, M, unpublished).

To address the biological relevance of the interplay of Cav-1 with influenza proteins we performed inhibition experiments with a dominant-negative Cav-1 mutant, knock-down by Cav-1 RNAi as well as competition experiments with M2 fusion proteins. We found, that the yield of human influenza virus progeny is affected by the presence/absence of Cav-1. The data suggest that Cav-1 can support the human influenza virus A life cycle. Pull-down and co-immunoprecipitation experiments were performed which showed binding of M2 and Cav-1.

## Results

### Influenza A virus titres are affected in MDCK Cav-1 knock-down cells

**Figure 1:**
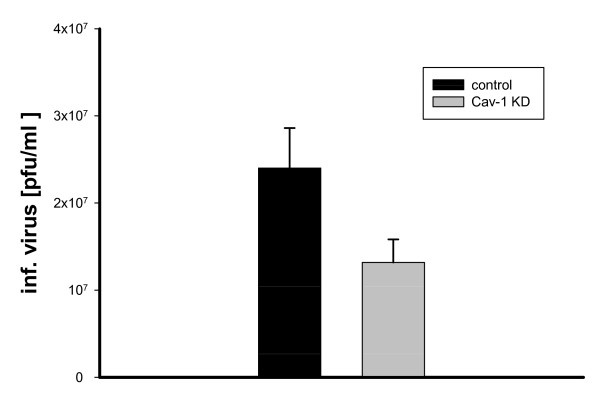
**Inhibition of influenza A/PR/8/34 multiplication in MDCK Cav-1 knock-down cells**. Titres of A/PR8 infected MDCK Cav-1 knock-down cells at day 13-17 after RNAi vector treatment and infection with influenza A/PR/8/34. Standard errors are depicted. Analysis using a paired t-test (n = 9) revealed that the 1,5 to 3 fold reduction in titres compared to MDCK control cells is statistically highly significant (p > 0.001).

We used MDCK (ATCC CCL-34), a canine kidney cell line commonly used in basic influenza virus research and vaccine production [16-19]. To elucidate the biological importance of Cav-1 in the influenza life cycle, MDCK cells were infected with a selectable retroviral Cav-1 RNAi vector carrying a puromycin-resistance gene (RVH1-Puro-Cav-1) as well as control RVH1-Puro alone [20]. We found that the Cav-1 content decreased gradually to 25% of the value in wild-type MDCK at 14-17 days post infection (d.p.i.) (data not shown). Next, Cav-1RNAi-MDCK cells exhibiting the lowest Cav-1 levels (day 17 p.i.), wt-MDCK or RVH1Puro-MDCK were chosen for infection experiments with influenza A virus (Fig. 1). A high m.o.i. of 10 was used to challenge the host system, as residual Cav-1 in knock-down cells may suffice to support influenza virus production upon infection at low m.o.i. Maximum titres of 4.5 × 10^7 ^pfu/ml were achieved from wild-type cells in a plaque assay. Strikingly, titres from Cav-1 knock-down MDCK cells were decreased up to to 32% of wild-type level. The infection experiments were repeated at different days post RNAi transfer (12, 15, 20 d.p.i) and with different m.o.i. (0.1, 1, 10). Notably, the experiments revealed similar results with an average decrease of influenza titres to 57.3% of wild-type levels (Fig. 1). The statistical analysis of nine independent experiments revealed that the 1.5-3 fold reduction in titres observed is highly significant (paired t-test, >99% confidence, p > 0,01) When cells stably transduced with control virus vector devoid of Cav-1 interfering sequences (RVH1puro) were infected with influenza A virus (m.o.i. = 10) titres of released virus was affected only marginally. Thus, we conclude that Cav-1 reduction in MDCK is correlated with a decrease in influenza virus progeny. This suggests, that Cav-1 directly or indirectly affects the human influenza virus life cycle in MDCK cells.

### A dominant-negative Cav-1 mutant decreases influenza A virus titres in MDCK cells

A dominant-negative Cav-1 mutant has been described which functionally inactivates caveolin-1 upon binding [21]. The mutant carries a F92A/V94A double mutation in the scaffolding domain (SD) of canine caveolin-1. Expression in rat adipose and COS-1 cells has been shown to interfere with the interaction of Cav-1 with the insulin receptor and impairs receptor function.

**Figure 2:**
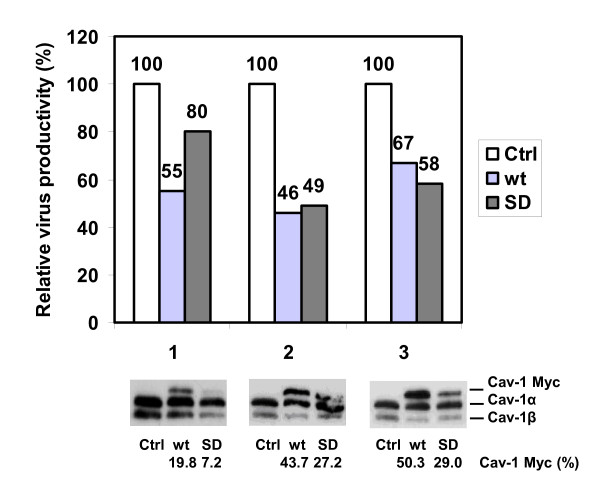
**Inhibition of influenza A/PR/8/34 multiplication in MDCK cells by means of a dominant-negative Cav-1 mutant**. Top: Relative titres of MDCK cells expressing myc-tagged dominant-negative caveolin-1 (SD), wild-type (wt) Cav-1 cDNA or mock-transfected cells (Ctrl) 24 h after infection with influenza A/PR8 (m.o.i. = 1) and normalisation to wt MDCK infection (100%). Results of three independent experiments are shown. Bottom: Immunodetection of endogenous, myc-tagged wild-type (wt) and mutant caveolin-1 (SD) in transfected MDCK. Relative protein levels are indicated (endogeneous Cav-1 = 100%). Cav-1 appears in the two known isoforms (Cav-1α 24 kDa; Cav-1β 21 kDa), the β-isoform is missing 31 aminoterminal residues of the Cav-1 protein.

To confirm our results from knock-down experiments, we investigated the effect of expression of the dominant-negative SD mutant and over-expression of wild-type caveolin-1 on virus production. Expression efficiency could be monitored easily, as endogenous and transfected, recombinant caveolins differ in their mobility in SDS-PAGE due to a C-terminal myc-tag (Fig. 2 bottom). Cav-1 appeared in two isoforms, with molecular weights of 21 and 24 kD, respectively [22,23]. Expression efficiencies ranged from 7-29% (SD) and 20-50% (wt-Cav-1) with respect to endogenous Cav-1 level. Provided that the myc-tag does not interfere with Cav-1 antibody detection and assuming a 1:1 interaction of SD mutant and endogenous Cav-1, sufficient competitor amounts should be available in successfully transfected cells. Next, transiently transfected MDCK and mock-transfected MDCK cells were infected with influenza A/PR/8/34 virus one day after transfection. 24 h later supernatants were used for titre determination (Fig. 2 top). To account for between-session-variations in cell culture, values were normalized to virus production from infected wt MDCK (100%). To exclude sensitivity of influenza infection to the actual transfection process, pEGFP-N1 transfected control cells were infected with PR8 virus in a control experiment. Strikingly, SD mutant expression in MDCK cells interfered with human influenza A virus replication and decreased the viral titres on average 1.6 fold to 62% of titres from wild-type MDCK (average of three independent experiments, standard deviation = ± 15,95). Compared to processed EGFP control cells, virus yield from Cav-1 wt- or SD-transfected MDCK cells was reduced considerably, which excludes that effects observed on virus production are derived from the transfection process (data not shown). This strongly suggests that interference with Cav-1 function in MDCK cells interferes with human influenza A virus replication. Interestingly, over-expression of wild-type Cav-1 also diminished influenza virus production, since viral titres reached only 56% ± 10.53 compared to non-treated MDCK (n = 3). Thus, surplus exogenous Cav-1 interferes with endogenous Cav-1 function, too. However, compared to the SD mutant twice the amount of Cav-1 wt is necessary to account for a comparable level of inhibition, as judged by Western Blot analysis.

### Competition with an influenza virus structural protein decreases influenza A virus production in MDCK cells

#### Search for putative Cav-1 interaction partners

**Figure 3:**
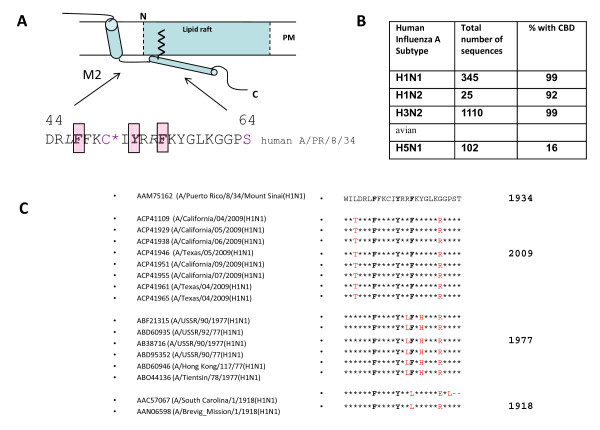
**Schematic representation of M2 domains and conservation of a putative caveolin-1 binding domain in human influenza A viruses**. **A**. For reasons of clearness, only a M2 monomer is indicated in the drawing. M2 tetramers function as an ion-pump (residing in a helical domain in the transmembrane region represented by cylinder 1). The C-terminal region is important for virus assembly and budding. A palmitoyl residue (jigsaw line) is linked to cysteine 50. The caveolin-1 binding domain resides in the loop and helical domain (cylinder 2) tilted perpendicularly with respect to the TM domain and is supposed to face the inner leaflet of the membrane. **B**. Conservation of a putative caveolin-1 binding domain. The core motif of the caveolin-1 binding domain (bold letters **F**47, **Y**52, **F**55) is highly conserved among most subtypes of human influenza A viruses (insert). **C**. Alignment of M2 (H1N1) sequences. The putative CBD core (bold) and adjacent sequences of influenza A viruses of pandemic H1N1 strains (2009 USA/Mexico, 1977 'Russian flu', 1918 'Spanish flu') were aligned to the M2 region (aa 41-65) of the Puerto Rico strain 8/1934. Conserved residues: asterisks *. Amino acid deviations: faint red.

In order to elucidate the molecular basis of the interaction we scanned influenza A virus proteins for putative binding motifs. Cav-1 binds to various cellular proteins like membrane receptors, soluble or membrane-associated molecules [24] as well as several viral proteins and exerts functions in localisation, transport and cellular signalling (Table 1). Signalling is preceded by phosphorylation of Cav-1, which initiates events leading either to activation of specific signalling pathways [21] or maintenance of signalling-competent, yet inactive complexes [24]. A specific, lumenal domain termed caveolin scaffolding domain (CSD, aa 82-101) which resides adjacent to the region of membrane insertion, is responsible for specific protein binding in the vast majority of cases [24,25]. Two consensus sequences have been identified in phage-display experiments and in the primary structure of Cav-1 binding partners which were termed caveolin binding domains (CBD) [26]. CBDs have been recognized in cellular [24] and viral proteins (Table 1). The consensus sequence comprises a run of 3 aromatic residues (W, F, Y) separated by a characteristic spacing (ΦxxxxΦxxΦ; ΦxΦxxxxΦ; where x stands for any amino acid and Φ for W, F, Y). Our screening for CBDs identified putative binding regions in HA, PB2 and M2 of influenza A virus. Especially, a region in the M2 channel protein turned out to be highly conserved among human influenza A viruses (Fig. 3B). The putative CBD overlaps with a loop/helical domain immediately following the M2 transmembrane region at the lumenal site of M2 (Fig. 3A). The CBD surrounds Cys 50, which is palmitoylated and faces the membrane allowing for insertion of the palmitoyl residue into the lipid bilayer. Thus, the CBD would be located favourably for interaction with the Cav-1 scaffolding domain [27,28]. Strikingly, compared to M2 of A/PR8/34 as a reference the CDB core motif (positions F47, Y52, F55) and immediately adjacent amino acid residues are completely conserved in 8 M2 sequences available for the pandemic influenza virus of swine origin A/2009 (H1N1) (Fig. 3C). Furthermore, motif conservation is observed in a former H1N1 strain appearing in 1977, but homology is restricted to the aromatic core and to a lesser extent to adjacent residues. Surprisingly, in M2 of influenza A/1918 the CBD motif is not conserved, as its third aromatic residue phenylalanine is changed to leucine, a residue commonly found in the M2 of avian influenza A viruses at that position (G.-V. Hemgård and M. Wirth, unpublished observation).

**Table 1:** Cav-1 interactions with viral proteins

Virus family (-*viridae*)	Virus	Protein	Protein function	Type of interaction with protein partner	Type of interaction with Cav-1	Reference
*Retro*	HIV-1	gp41	Transmembrane, fusion	Binding to CBD in HIV-1, but not HIV-2 or SIV	Binding to CSD*	(Benferhat et al., 2008; Hovanessian et al., 2004)
	
	HIV-1	gp41	Transmembrane, fusion	Binding to six-helix bundle	Binding to CSD	(Huang et al., 2007)
	
	HIV-1	Not known		Not known	Cav-1 membrane insertion domain	(Llano et al., 2002)
	
	MLV-amphotropic, ecotropic	MA-Gag	Matrix, associates with membranes, link between capsid, plasma membrane, and viral membrane proteins	Binding mediated by a CBD in MA, interaction locates MA to lipid rafts domains in PM	Interaction with CSD*†	(Beer and Wirth, 2004; Yu et al., 2006)

*Corona*	SARS	ORF3a	Not known, Functioning in Golgi localization?	Binding to several CBDs	Not known, interaction with CSD likely	(Padhan et al., 2007)

*Orthomyxo*	Influenza A virus human	M2	Early phase: Ion channel, viroporin Late Phase: matrix, virus assembly and budding	Binding. Protein regions presumably CBD aa47-55	Binding to CSD*† Binding to CSD‡	This investigation Zou et al. 2009
	
	Influenza A virus human	HA	Receptor binding	Colocalization in perinuclear regions		(Scheiffele et al., 1998)

*Paramyxo*	RSV	?	?	Colocalization with internal viral filaments, colocalization at lipid rafts	Binding not specified, redistribution of Cav-1 after phosphorylation	(Brown et al., 2002; Brown, Rixon, and Sugrue, 2002; McDonald et al., 2004)

*Reo*	Rotavirus	NSP4	Ion channel formation, ER and caveolae localization, important for morphogenesis	Binding aa114-135 (enterotoxic peptide) amphipatic helix at the C-terminus	Binding and colocalization, 2 independent binding sites at the N-terminus (aa2-22)and C-terminus (aa161-178) identified, influence on localization or transport?	(Mir et al., 2007; Parr et al., 2006; Storey et al., 2007)

*Pull-down experiments with biotinylated CBD-peptides

^†^Co-immunoprecipitation

^‡ ^ELISA

#### Competition of Cav-1 binding with M2 affects production of influenza A/PR/8/34

**Figure 4:**
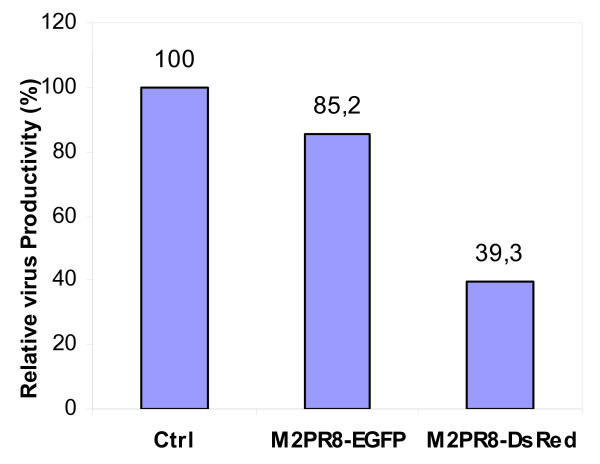
**M2 competition decreases influenza A/PR/8/34 titres in MDCK cells**. Titres from infected MDCK cells transiently transfected with M2 fusion vectors or mock-Cells were infected with influenza A/PR/8/34 virus 1 d after transfection, infectious titres were determined 1 d later using plaque assays. The average of two independent experiments is shown.

These hints prompted us to investigate the effect of M2 over-expression on the influenza A virus life cycle in MDCK cells. We hypothesized, that surplus M2 fusion protein may reduce the concentration of available, functional Cav-1 by complexing. To monitor M2 protein levels and localization we generated mammalian expression vectors containing cDNAs for fusion proteins of M2 (A/PR/8/34) with desRedExpress, a red fluorescent, tetrameric protein (pM2PR8DsRed) or EGFP (enhanced green fluorescent protein) (pM2PR8_EGFP) and transfected purified DNA into MDCK cells. Expression levels and localization of the fluorescent proteins were followed 1 and 2d after transfection. The transfection efficiency (ratio of fluorescent/nonfluorescent cells) ranged between 10 and 15%. M2 fluorescent fusion proteins initially were found in the cytoplasma and started to localize at the plasma membrane at day 1 post transfection. As expected M2DsRed and M2EGFP localization did not differ from localization of M2 after infection with A/PR8/34, except that upon over-expression M2 fusion proteins partially stacked in juxtanuclear regions (data not shown). Next, we carried out mixed transfection/infection experiments. For that purpose M2dsREd, M2EGFP and mock-transfected MDCK cells were infected with influenza A/PR/8/34 one day after transfection. 24 h later supernatants were collected and processed for titre determination (Fig. 4). Interestingly, M2 expression in infected MDCK decreased viral titres to 40% (M2DsRed) and 85% of (M2EGFP) of the level of non-transfected cells. Thus, M2 over-expression interferes with human influenza virus propagation, presumably by competing with endogeneous M2 for Cav-1 interaction.

### The M2 matrix protein of human influenza A interacts with Cav-1

**Figure 5:**
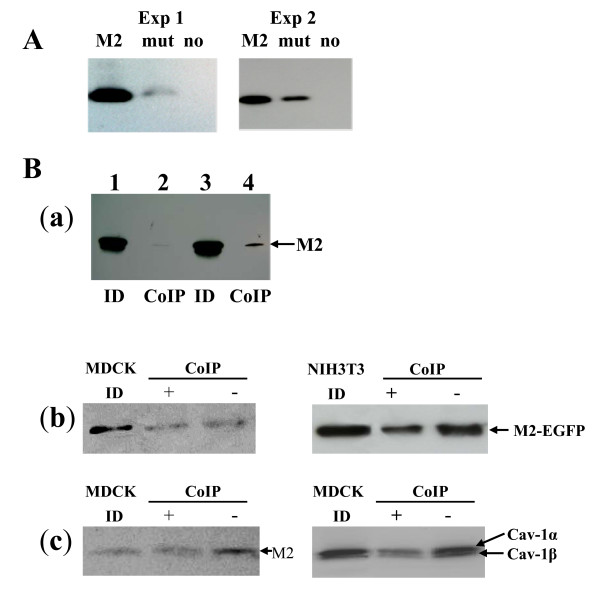
**Specificity of Cav-1 binding to M2 of human Influenza A virus and the participation of cholesterol**. **A**. Pull-down experiments using biotinylated peptides representing the wt M2 CBD or a mutated sequence where aromatic residues in the CBD were changed to alanine. For Cav-1 detection after Western Blot a rabbit polyclonal antibody was used. **B**. Co-immunoprecipitation (Co-IP) experiments with pM2PR8-EGFP transfected or A/PR8 virus-infected cells. a. Lysates of pEP24c-transfected MDCK cells were processed for co-immumoprecipitation (polyclonal anti-Cav-1 antibody) followed by Western Blot detection of M2 (14C2). b. (Co-IP) of lysates of pM2PR8-EGFP transfected MDCK cells with or without cholesterol depletion by addition of methyl-β-cyclodextran (MβCD) (+) or mock-treatment (-) by rabbit polyclonal anti-Cav-1 antibody (Co-IP) and monoclonal mouse anti-EGFP antibody (indirect M2 detection) were used. MDCK ID: Lysates from transfected cells processed for immunodetection only. c. Lysates of A/PR8 Virus infected cells were processed for immunodetection (MDCK ID) or co-immunoprecipiation (CoIP) after cholesterol depletion with MBCD (+) or mock-treatment (-). Left panel: CoIP using rabbit anti-Cav-1 pAb and detection with anti-M2 antibody (14C2). Right panel: Co-IP: mouse anti-EGFP mAb.Detection: rabbit anti-Cav-1 pAb.MDCK ID: lysates from infected MDCK cells processed for immuno-detection.

To verify the predicted M2/Cav-1 binding, pull-down as well as co-immunoprecipitation experiments were carried out. For pull-down experiments, biotinylated peptides carrying the putative CBD of M2 or a mutant CBD with alanines instead of the motif's core aromatic residues were incubated with cell lysates and complexes were processed as specified in Material and Methods (Fig. 5A). Results from two independent experiments show that the M2 CBD-peptide indeed pulls down caveolin-1, while the alanine-CBD mutant exhibits a strongly reduced tendency to interact with Cav-1. These results indicate that M2 of influenza A/PR/8/34 indeed exhibits at least one caveolin-binding domain.

To confirm data of the pull-down experiments, co-immunoprecipitation experiments were performed using NIH3T3 or MDCK cells after transfection of pEP24c, an expression vector containing M2 PR/8 [29] or a vector harbouring fusion protein of M2 with fluorescent marker EGFP. 24 h later cell lysates were prepared in the presence of octylglucoside, a detergent that disrupt lipid rafts, as described previously [13]. In the first series of experiments, polyclonal anti-Cav-1 antibodies were used to pull-down Cav-1 complexes from lysates. Precipitated complexes were probed for the presence of M2 after Western Blot and immunostaining. In these experiments, the Cav-1 antibodies clearly pulled down a complex that contained a M2 from pEP24c transfected MDCK cells (Fig. 5B, a) or the M2 fusion protein from pM2PR8-EGFP transfected MDCK or NIH3T3 cells (Fig. 5B, b) as well as infected MDCK cells (Fig. 5B, c left panel). In the second experimental setting, vice versa, monoclonal anti-EGFP antibodies were used to precipitate M2 binding partners and a rabbit anti-Cav-1 antibody was used to probe for the presence of caveolin (Fig 5B, c right panel). These types of experimental settings identified M2 complexed with Cav-1 and vice versa in both cell lines, NIH3T3 and MDCK. Thus, the results suggest that M2 has the capability to interact directly or indirectly with caveolin-1 in different cell lines. With respect to the type of interaction, it is notable, that caveolin-1 as well as M2 have been reported to bind cholesterol via cholesterol specific recognition domains [30,31]. This prompted us to investigate, whether cholesterol is involved in the M2/caveolin-1 interaction. For that purpose methyl-β-cyclodextrin (MβCD) was used to deplete cell lysates from cholesterol before co-immunoprecipatation (Fig. 5B b and 5c). Interestingly, in pM2PR8-EGFP transfected NIH3T3 cells as well as in PR8 virus-infected MDCK cells, signals from co-immunoprecipated proteins decreased to a certain extent, if cholesterol was removed from the lysate before pull-down. These findings imply, that cholesterol seems to support the interaction of M2 with caveolin-1.

## Discussion

Viruses recruit the cellular machinery to support their own multiplication and elicit an early host response to overcome the unwanted viral invaders. In our contribution we investigated the ability of caveolin-1, a multifunctional protein, to interact with components in the influenza A virus life cycle and to interfere with influenza A virus production. Cav-1 represents an organizing element at the plasma membrane and serves on localization and accumulation of proteins in lipid rafts and transmission of signalling events [24]. Furthermore, the protein contributes to intracellular cholesterol transport and has been identified as the main determinant of caveolae, invaginations of the plasma membrane used for entry of molecules and particles into the cell.

Based on previous findings of Cav-1 involvement in the late retroviral life cycle [13] we investigated the influence of Cav-1 on human influenza A/PR/34 (H1N1) virus multiplication in inhibition experiments. It is crucial for our investigation, that influenza virus entry does not occur via caveolae, but can be mediated by chlatrin-dependent endocytosis or another, not-defined pathway independent of chlathrin-coated pits [32-34]. For example, it has been shown, that a Cav-1 dominant-negative mutant does not affect the entry of influenza virus [32]. The findings are a prerequisite to exclude artifacts that may arise from insufficient entry due to Cav-1 depletion in inhibition experiments. Applying different methods to impair or inhibit Cav-1 function in MDCK, a knock-down procedure, a dominant-negative Cav-1 mutant as well as competition experiments with M2 fusion proteins, we could show that Cav-1 influences human influenza A virus propagation. Inhibition methods have their limitation, e.g., we noticed that Cav-1 RNAi-mediated knock-down resulted in diminution of Cav-1 expression levels in MDCK cells to 25% of Cav-1 wild-type level at the most. Concomitantly, virus yield from these cells decreased 2-3 fold of virus levels observed from wild-type or RNAi-vector treated MCDK cells. Unfortunately, the effect of complete absence of Cav-1 on human influenza A virus production in MDCK cells could not be investigated, as further reduction of Cav-1 levels cannot be achieved with the retroviral RNAi system used [20]. This question may be answered in a Cav-1 (-/-) MDCK cell line, which yet has to be established.

Data from knock-down experiments in MDCK were supplemented by transfection of a dominant-negative Cav-1 mutant as well as Cav-1 over-expression, which decreased viral yields by 38-44%. The results are reminiscent of experiments of Nystrom et al. who observed impairment of the insulin signalling pathway upon expression of both, the dominant-negative Cav-1 mutant and the over-expressed Cav-1 wt cDNA as well [21]. Finally, competition with M2 fusion proteins impaired virus replication, too.

Taken together Cav-1 supports virus multiplication in MDCK, but the cellular pathway directing this Cav-1 property is not known. It is conceivable, that the cellular protein level of Cav-1 is important for the outcome, as it has been suggested for Cav-1 involvement in the insulin pathway [21].

Hints for the molecular basis of influenza virus/Cav-1 interaction may come from other viruses which co-opt Cav-1. It is evident that individual stages in the various viral life times are affected and different roles are allocated to Cav-1 as well (Table 1). For example, the CBD region in the HIV-1 gp41 transmembrane protein can permeate membranes and is supposed to augment the fusion step upon virus entry. Remarkably, respiratory syncytial virus (RSV), induces Cav-1 phosphorylation, which results in intracellular relocation of proteins during the paramyxovirus life-cycle. In several cases, Cav-1 functions in positioning of viral proteins to intracellular membranes (Rotavirus, SARS) or specialised regions of the plasma membrane like lipid rafts (retrovirus MLV).

To understand the molecular basis of the Cav-1 contribution to influenza A virus propagation we focussed on Cav-1 interactions mediated by the caveolin-scaffolding domain (CSD, aa 81-102) [25]. Database searches and subsequent peptide pull-down assays in combination with co-immunoprecipitation experiments suggested binding of caveolin-1 to M2 presumably to a motif in the M2 protein fitting the CBD consensus [26]. Strikingly, the motif is shared in M2 of nearly all human influenza A viruses. M2 functions within the viral life cycle as a viroporin with proton channel activity that is crucial in the entry phase [1] and as a maturation cofactor in virus budding. The cytoplasmic tail is implicated in M1 binding and facilitates virus assembly and production [2-4,35]. Furthermore, Schroeder et al. showed that avian M2 is a cholesterol-binding protein [31]. Most avian influenza A viruses contain two cholesterol recognition motifs (CRAC I, CRAC II) in close vicinity to the transmembrane domain in the cytoplasmic region of M2 [31,36]. Thus, cholesterol-binding and palmitoylation in combination with a short transmembrane region may direct M2 to the raft periphery in membranes and may promote clustering and merging of rafts which is then followed by the pinching-off of avian viruses [31]. With this model for avian influenza virus in mind it is conceivable that the interaction of Cav-1 with M2 could direct the protein into the vicinity of lipid rafts in human influenza A virus infection. This view may be supported by different observations: Firstly, we observed that the caveolin-1 binding domain is present in M2 of most human influenza A virus strains and overlaps with a CRAC motif for cholesterol binding. Such a high degree of evolutionary conservation generally suggests a constant selective pressure to preserve a specific function in the viral life cycle. Secondly, Cav-1 itself binds cholesterol via a region in the caveolin scaffolding domain [30]. Notably, to some degree Cav-1 binding to M2 is sensitive to the cholesterol depletion (this investigation). Preliminary results of mutagenesis as well as localization experiments indicate a certain role of the M2-CBD in M2 transport and localization (unpublished observations). Taken together our results demonstrate, that Cav-1 exerts an influence on influenza A virus replication and data imply that the binding of Cav-1 to the matrix protein M2 is involved. However, which function or pathway in MDCK cells actually is triggered via Cav-1 interaction with M2, remains to be determined.

## Conclusion

The appearance of the aggressive bird influenza (H5N1), the 2009 outbreak of a pandemic influenza (H1N1) of swine influenza origin, and the recent occurrence and rapid dissemination of oseltamivir-resistant human influenza strains are motors that have accelerated the search for new antiviral targets and agents within the last time [37-39]. The investigation of cellular mechanisms involved in 'early' and 'late' viral processes and the identification of cellular actors provides a means to interfere with viral strategies. With this respect, the observed Cav-1/M2 interplay may represent a new, conserved target for e.g. therapeutic intervention with circulating and newly emerging strains of human influenza A virus. Thus, application of high-throughput screening of compound libraries will follow target identification and may result in a new antiviral agent, as exemplified for a cellular target involved in the late retroviral life cycle [40].

When this manuscript was in preparation Zhou et al. reported binding of a cytoplasmic fragment of M2 from human influenza to Cav-1 in an *in vitro *assay based on a Cav-1 protein fragment expressed in E. coli and CBD-dependent perinuclear co-localization upon expression in CHO cells [41]. However, no experiments on the functional importance of M2/Cav-1 were performed in this investigation.

## Materials and methods

### Cells and viruses

MDCK Madin-Darby canine kidney (ATCC CCL-34) and NIH 3T3 (ATCC CRL-1685) were maintained in Dulbecco's modified Eagle's medium (DMEM) supplemented with 10% fetal calf serum and 2 mM L-Glutamine at 37°C in 5% CO_2_. Influenza A/Puerto RicoR/8/34 (H1N1, Mount Sinai strain) virus was generously provided by Stephan Ludwig (Virology, ZMBE, Muenster, Germany).

### Chemicals

BCA protein assay kit (Pierce) Methyl-β-cyclodextrin (MβCD, Sigma), octyl glucoside (Applichem) and other chemicals were of the highest grade commercially available.

### Plasmids

pCav-1 wt (myc-tagged canine Cav-1 cDNA in pCIS2) and pCav-1 SD (point mutations F92A V94A in scaffolding domain) are described elsewhere [21]. pM2PR8-EGFP and pM2PR8-dsRED were constructed by PCR-cloning of M2 (A/PR/8/34/(H1/N1) into BamHI/AgeI linearized pEGFP-N1 and pDsRed-Express-N1 (Clontech), respectively. M2 identity was verified by DNA sequencing. pEP24c (M2 cDNA) [29]. pRVH1-Puro-Cav-1 and pRVH1-Puro [20] are described elsewhere.

### Antibodies

Rabbit anti-caveolin 1 polyclonal antibody (pAb), mouse anti-caveolin 1 monoclonal antibody (mAb) mouse anti-EGFP mAb (JL-8) (all BD Transduction Laboratories) mouse anti-Influenza A virus M2 monoclonal antibody (14C2, ABR) were used according to the suggestions of the supplier.

### Infections

Infections with Influenza A/PR8/34 were performed in the presence of trypsin (1-2 μg/ml) at a multiplicity of infection (m.o.i) of 0.2-10 for 2 h at 37°C. Virus stocks were prepared from supernatants of MDCK cell cultures one day post infection (1 d.p.i.).

### Transfection

Plasmids were transfected into cells via Lipofectamine 2000 (Invitrogen or by calcium phosphate transfection [42].

### Lysis of cells

Lysates were prepared as described previously [13].

### Plaque Assay

Influenza A/PR/8/34 titre was determined by plaque assay on MDCK cells. PBS- washed MDCK were inoculated with 500 μl of virus dilution for 1-2h at 37°C. Cells were covered with 2 ml of MEM medium containing 1% purified agar (Oxoid, England) and 1-2 μg/ml trypsin (Sigma). After three days incubation at 37°C, plates were stained with 0.03% neutral red staining to facilitate plaque counting.

### Pull-down experiments

20 μM biotinylated peptide encompassing either the conserved CBD within human influenza M2 (Bio-β-Ala-LDRLFFKCIYRFFKHGL-amid) or a mutant where the CBD core motif is exchanged by alanine residues (Bio-β-Ala-LDRLAFKCIYRFAKHGL-amid) were inoculated with 50 μl NIH3T3 cell lysate (2 ml, T75 flask) for 90 min. Complexes were immobilized using 10 μl streptavidin coated paramagnetic microbeads and μ column (Miltenyi). Washed samples were eluted with 1× sample buffer preheated at 95°C for 2 min and 15 μl out of 70 μl eluate were separated by SDS PAGE, blotted to PVDF membrane and probed with rabbit anti-caveolin-1 antibody.

### Co-immunoprecipitation

Cell lysates were incubated with rabbit anti-caveolin-1 antibody (1:2000) or mouse anti-EGFP antibody (1:100) at 4°C for 1 h, treated with 20-50 μl protein A- or G microbeads (Miltenyi) at 4°C for 1 h, and processed as described previously [13]. To deplete cholesterol, cell lysates were treated with 10-20 mM MβCD at room temperature for 1 h before co-immunoprecipitation.

### SDS-PAGE and Western Blot

Protein concentrations were determined using the BCA kit (Pierce). 5 μg total protein was separated on a vertical 12% separating gel. Subsequently, proteins were transferred to PVDF membranes using a Transblot™ Semi-dry transfer cell (Bio-Rad). After blocking for 1 h (0.2% CA blocking reagent, Applichem) immunostaining was performed with primary antibody followed by 4 washing steps (TBS 0,02% Tween 20) and addition of the secondary antibody at appropriate dilution. The blots were developed with chemoluminescent substrate (Supersignal Femto West, Supersignal Pico West, Pierce). The band intensities were quantified using QuantityOne software (Bio-Rad) and ImageJ.

### Inhibition and competition experiments

#### Generation of Cav-1 knock-down MDCK using retrovirally mediated RNAi

The recombinant retroviral vectors were produced from 293T triple transfection of pCMV1MLVGP1, encoding MLV *gagpol*, pVSV-G, pRVH1-Puro-Cav-1 encoding a shRNA for Cav-1 inhibition and a puromycin resistance gene, as described [20]. For knock-down MDCK (60%-80% confluency) were infected with the respective shRNA retroviral vectors in the presence of 4 mg/ml polybrene for 48 hours. Puromycin-resistant clones were pooled and further analysed 10-27 days after infection.

#### Inhibition using a dominant-negative Cav-1 mutant

Plasmids pCav-1 wt or pCav-1 SD (Scaffolding domain mutant) were transiently introduced into MDCK, NIH3T3 or MEF 3T3 KO cells using lipofectamine 2000.

#### Competion with M2 fusion proteins

Plasmids pM2PR8_EGFP or pM2PR8DsRed were transiently transfected into MDCK cells by lipofection. The cells were infected with influenza A/PR/8 virus 1 day after transfection. Virus titres were determined from supernatants after additional 24 h of incubation at 37°C.

## Competing interests

The authors declare that they have no competing interests.

## Authors' contributions

GVH and MW did the data base analyses. LS and GVH performed the co-immunoprecipi-tations. MW carried out pull-down the experiments. LS, GVH and SAS carried out the influenza infection experiments. LS and SAS performed the inhibition and competition experiments and were engaged in plasmid cloning. MW designed the study and supervised the experiments. MW drafted and finalized the manuscript. All authors read and approved the manuscript.
